# Piwi-like 1 protein expression is a prognostic factor for renal cell carcinoma patients

**DOI:** 10.1038/s41598-018-38254-3

**Published:** 2019-02-11

**Authors:** Christine G. Stöhr, Sandra Steffens, Iris Polifka, Rudolf Jung, Andreas Kahlmeyer, Philipp Ivanyi, Florian Weber, Arndt Hartmann, Bernd Wullich, Sven Wach, Helge Taubert

**Affiliations:** 1 Institute of Pathology, University Hospital Erlangen, FAU Erlangen-Nürnberg, Erlangen, Germany; 2 Present Address: Clinic for Urology, University Hospital Muenster, Muenster, Germany; 3 Department of Urology, Hannover Medical School, Hannover, Germany; 4 Department of Urology and Pediatric Urology, University Hospital Erlangen, FAU Erlangen-Nürnberg, Erlangen, Germany; 5 Department of Hematology, Hemostasis, Oncology and Stem Cell Transplantation, Hannover Medical School, Hannover, Germany; 6 Institute of Pathology, University Regensburg, Regensburg, Germany

## Abstract

The Piwi-like genes belong to the Argonaute gene family and are conserved in plants, animals and humans. In addition to their essential role in the germ line and as stem cell-associated genes, Piwi-like proteins play a role in different cancer types but have yet to be studied in renal cell carcinoma (RCC). We investigated tissue micro arrays (TMAs) with tumor samples of two independent cohorts of RCC patients (N = 265 and N = 345); we used immunohistochemistry to assess the protein expression of Piwi-like 1. Applying an immunoreactive score (IRS), we found Piwi-like 1 positivity (IRS > 0) in 28.3% and 14.8% of the tumors in cohorts 1 and 2, respectively. Piwi-like 1 positivity was correlated with Fuhrman grade, tumor stage and the presence of distant metastasis (P < 0.005). Moreover, in univariate and multivariate analyses (adjusted to Fuhrman grade and tumor stage), Piwi-like 1 positivity was associated with a shorter cancer-specific survival in the patients in the second cohort. In addition, Piwi-like 1 expression allowed to further distinguish the RCC patients with high Fuhrman grade, high tumor stage, distant metastasis or high pre-operative levels of C-reactive protein, as Piwi-like 1 positivity was associated with a shorter cancer-specific survival in both cohorts. Our data encourage further investigations to enlighten the role of Piwi-like 1 and its function as a marker of poor prognosis in RCC patients.

## Introduction

The Piwi gene was first identified as P-element-induced wimpy testis mutation that abolished germline stem cell division in Drosophila melanogaster^1^. The Piwi-like genes belong to the Argonaute gene family and are conserved in plants, animals and humans^2,3^. In humans, four members (Piwi-like 1, −2, −3, −4) have been identified^4^. Piwi-like genes/proteins are essential for stem cell self-renewal and are expressed predominantly in the germline^5–7^. Piwi-like proteins catalyze an amplification loop (ping-pong cycle) of small RNAs, the so-called Piwi-interacting RNAs (piRNAs). Both piRNAs and Piwi-like proteins function as a Piwi-ribonucleoprotein complex at transposon repression through target degradation and epigenetic silencing^8,9^. In addition to their expression in the germ line, an increased expression in different tumors has been reported^10^. The detection of Piwi-like proteins has been shown to be associated with clinical parameters and/or poor prognosis in many cancers^10–12^. Furthermore, Piwi-like 1 protein overexpression has been linked to cell physiological parameters in tumors, e.g., to cell proliferation in gastric cancer^13^. There are two studies of Piwi-like mRNA expression levels in RCC patients^14,15^. We showed that Piwi-like 1 mRNA levels were higher in younger patients in comparison to older patients^14^. Illiev *et al*. found that decreased mRNA levels of Piwi-like 1, −2 and −4 were associated with an increased tumor stage and a worse overall survival in RCC patients^15^. However, the expression of Piwi-like proteins has not yet been studied in RCC.

## Results

### Piwl-like 1 expression and correlation with clinico-pathological parameters

#### Cohort 1

In the cohort 1 (N = 265), we noted 190 cases (71.7%) without and 75 cases (28.3%) with Piwi-like 1 expression (Suppl. Table 1; Table 1).

**Table 1:** Clinico-pathological data for RCC cohorts.

Clinico-pathological parameters	Patients cohort 1 (%)	Patients cohort 2 (%)
**Total**	265	345
**Morphology**		
clear cell	198 (74.7)	274 (79.4)
papillary	37 (14.0)	38 (11.0)
chromophobe	21 (7.9)	25 (7.3)
others	7 (2.6)	7 (2.0)
unknown	2 (0.8)	1 (0.3)
**Gender**		
females	85 (32.1)	119 (34.5)
males	180 (67.9)	226 (65.5)
**Age (years)**		
range	22.5–88.3	23.0–92.0
mean	62.0	64.2
median	62.9	66.0
**Tumor stage**		
pT1	67 (25.3)	215 (62.3)
pT2	106 (40.0)	35 (10.2)
pT3	84 (31.7)	84 (24.3)
pT4	7 (2.6)	1 (0.3)
unknown	1 (0.4)	10 (2.9)
**Tumor stage grouped**		
pT1 + pT2	173 (65.3)	250 (72.5)
pT3 + pT4	91 (34.3)	85 (24.6)
**Fuhrman grade**		
G1	34 (12.8)	40 (11.6)
G2	193 (72.9)	191 (55.3)
G3	34 (12.8)	102 (29.6)
G4	1 (0.4)	10 (2.9)
unknown	3 (1.1)	2 (0.6)
**Fuhrman grade grouped**		
G1 + G2	227 (85.7)	231 (66.9)
G3 + G4	35 (13.2)	112 (32.5)
**Tumor grade**	n.d.	
G1	n.d.	41 (11.9)
G2	n.d.	223 (64.6)
G3	n.d.	80 (23.2)
unknown	n.d.	1 (0.3)
**Lymph node metastasis**		
N0	229 (86.4)	69 (20.0)
N1/2	35 (13.2)	6 (1.7)
NX	0	150 (43.5)
unknown	1 (0.4)	120 (34.8)
**Distant metastasis**		
M0	200 (75.5)	261 (75.7)
M1	55 (20.8)	84 (24.3)
MX	9 (3.4)	0
unknown	1 (0.4)	0
**Survival/observation time**		
range	0–144.0	1–144.0
mean	62.7	45.2
median	62.1	38.0
**OS**		
alive	153 (57.7)	251 (72.8)
dead	112 (42.3)	94 (27.2)
**CSS**		
alive	164 (61.9)	311 (90.1)
dead	97 (36.6)	34 (9.9)
unknown	4 (1.5)	0

Regarding the Piwi-like 1 IRS, there was no correlation with age, gender, tumor histology, tumor size or survival status (OS and CSS), but there was a significant positive correlation with Fuhrman grade (r_s_ = 0.201), lymph node metastasis (r_s_ = 0.276), distant metastasis (r_s_ = 0.248), microvascular invasion (r_s_ = 0.199), collecting duct invasion (r_s_ = 0.203) (all P ≤ 0.001) and tumor stage (r_s_ = 0.163; P = 0.008). We also detected a negative correlation with survival time (r_s_ = −0.172; P = 0.005).

To further assess these findings, we grouped the data for a cross tables analysis (Chi^2^ tests). Piwi-like 1 protein expression, as detected by IHC, was grouped as negative (IRS = 0) and positive (IRS > 0). We detected Piwi-like 1 protein expression more often in the tumor stages T3 + T4 than in T1 + 2 (P = 0.001), in the high Fuhrman grades 3 + 4 than in 1 + 2 (P < 0.001), in patients with microvascular invasion compared to patients without (P = 0.002), in patients with collecting duct invasion compared to patients without (P = 0.004), in patients with lymph node metastasis compared to patients without (P < 0.001), and in patients with distant metastasis than in patients without distant metastasis (P < 0.001).

#### Cohort 2

In cohort 2 (N = 345), we detected 294 cases (85.2%) without and 51 cases (14.8%) with Piwi-like 1 expression (Suppl. Table 1; Table 1). We found no correlation of the Piwi-like 1 IRS with age, gender, tumor histology, microvascular invasion or lymph node metastasis, but a significant positive correlation with Fuhrman grade (r_s_ = 0.174; P = 0.001), survival status (CSS; r_s_ = 0.185; P = 0.001), distant metastasis (r_s_ = 0.153; P = 0.005), and tumor stage (r_s_ = 0.132; P = 0.027), and a negative correlation with survival time (r_s_ = -0.197; P < 0.001).

To verify these findings further, we again grouped the data for a cross tables analysis (Chi^2^ tests). Piwi-like 1 positivity occurred more often in the high Fuhrman grades 3 + 4 than in 1 + 2 (P = 0.011), in the tumors of patients who died from a tumor specific cause (P < 0.001), and in tumors from patients with distant metastasis compared to patients without distant metastasis (P = 0.002).

Altogether, in both cohorts, we validated a positive correlation of Piwi-like1 protein expression with higher Fuhrman grades, higher tumor stage and the occurrence of distant metastasis and a negative correlation with survival time.

### Piwi-like 1 protein expression and survival

#### All the RCC patients

**Figure 1:**
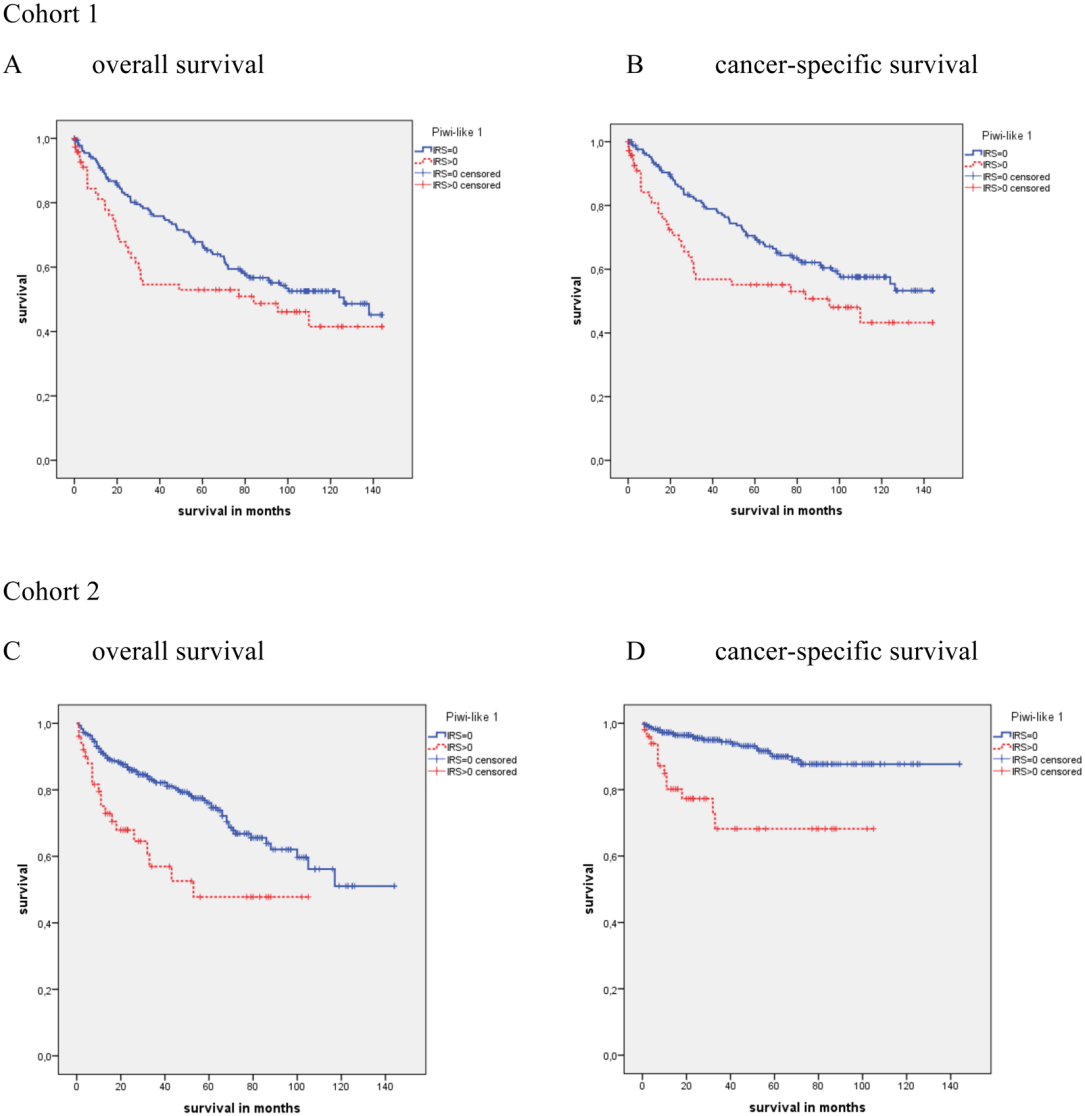
Kaplan-Meier analysis for overall (OS) and cancer specific survival (CSS) of all RCC patients in cohort 1 and cohort 2. In cohort 1 no significant association of Piwi-like 1 positivity with OS (P = 0.096) but a significant association with CSS (P = 0.032) was observed. In cohort 2 patients with Piwi-like 1 positive tumors showed a shorter OS (P = 0.001) and CSS (P < 0.001) than patients with Piwi-like 1 negative staining of their tumors. In this and all further survival analyses an IRS = 0 was considered as negative and an IRS > 0 as positive staining.

In cohort 1, by the Kaplan-Meier analysis no significant association of Piwi-like 1 positivity with OS (P = 0.096) was observed, but a significant association with CSS (P = 0.032) was detected. The mean CSS time was 81.1 months for patients with Piwi-like 1 positive tumors vs. 101.5 months for patients with Piwi-like 1 negative tumors (Fig. 1). An univariate Cox’s regression analysis revealed that Piwi-like 1 expression was associated with an 1.59-fold increased risk of cancer-specific death (CSD) (P = 0.033) but not with a significantly increased risk of death from any cause (P = 0.098; Table 2). In the multivariate Cox’s regression analysis (adjusted to Fuhrman grade and tumor stage), Piwi-like 1 protein expression in the tumor was not identified as an independent predictor of OS or CSS (Table 2).

**Table 2:** Univariate and multivariate Cox’s regression hazard analyses for all patients.

	Cohort 1						Cohort 1					
	OS			CSS			OS			CSS		
	univariate			univariate			multivariate			multivariate		
	RR	95%CI	P	RR	95%CI	P	RR	95%CI	P	RR	95%CI	P
**Piwi-like 1**												
IRS > 0 vs. IRS = 0	1.41	0.94–2.12	0.098	**1**.**59**	1.04–2.44	0.033	1.26	0.83–1.90	0.270	1.45	0.94–2.23	0.093
**Fuhrman grade**												
G3 + 4 vs. G1 + 2	**4**.**27**	2.71–6.72	<0.001	**4**.**57**	2.82–7.39	<0.001	**2**.**70**	1.65–4.41	<0.001	**2**.**57**	1.53–4.31	<0.001
**Tumor stage**												
T3 + 4 vs. 1 + 2	**3**.**16**	2.17–4.59	<0.001	**4**.**03**	2.69–6.04	<0.001	**2**.**46**	1.64–3.70	<0.001	**3**.**20**	2.07–4.95	<0.001
	**Cohort 2**						**Cohort 2**					
	**OS**			**CSS**			**OS**			**CSS**		
	**univariate**			**univariate**			**multivariate**			**multivariate**		
	**RR**	**95%CI**	**P**	**RR**	**95%CI**	**P**	**RR**	**95%CI**	**P**	**RR**	**95%CI**	**P**
**Piwi-like 1**												
IRS > 0 vs. IRS = 0	**2**.**24**	1.36–3.69	0.001	**4**.**37**	2.15–8.86	<0.001	**1**.**81**	1.08–3.01	0.024	**3**.**09**	1.48–6.44	0.003
**Fuhrman grade**												
G3 + 4 vs. G1 + 2	**3**.**26**	2.15–4.93	<0.001	**6**.**85**	3.18–14.77	<0.001	**2**.**62**	1.70–4.05	<0.001	**5**.**71**	2.50–13.06	<0.001
**Tumor stage**												
T3 + 4 vs. 1 + 2	**3**.**17**	2.10–4.78	<0.001	**2**.**80**	1.41–5.56	<0.001	**2**.**64**	1.74–4.01	<0.001	1.96	0.97–3.98	0.060

Significant values are marked in bold face.

In cohort 2, patients with Piwi-like 1 positive tumors showed a shorter OS and CSS. The mean OS time was 60.5 months for patients with Piwi-like 1 positive tumors vs. 101.7 months for patients with Piwi-like negative tumors (P = 0.001; Fig. 1). Concerning CSS, the patients with Piwi-like 1 positive tumors had a mean survival of 76.4 months vs. 131.9 months for the patients with Piwi-like 1 negative tumors (P < 0.001; Fig. 1). The univariate Cox’s regression analysis revealed that Piwi-like 1 positivity was associated with a 2.24-fold risk of death (P = 0.001) and a 4.37-fold increased risk for CSD (P < 0.001; Table 2). The multivariate Cox’s regression analysis (adjusted to Fuhrman grade and tumor stage) revealed that Piwi-like 1 positivity was an independent prognostic factor for OS (RR = 1.81; P = 0.024) and for CSS (RR = 3.09; P = 0.003; Table 2). However, when we included the presence of distant metastasis in the model, the tumor stage and Piwi-like 1 expression were no longer independent predictors of OS and CSS (data not shown).

#### Stratification according to histological subtype

Since it is known that tumor histology represents a major risk factor for outcome, we separately examined clear cell RCC, the most common histologic subtype.

Cohort 1 (N = 198) consisted of 139 patients (70.2%) with Piwi-like 1 negative clear cell RCC tumors and 59 patients (29.8%) with Piwi-like 1 positive clear cell RCC tumors. Patients with Piwi-like 1 positive clear cell RCC exhibited an average CSS of 78.6 months compared to. 94.2 months for patients with Piwi-like 1 negative tumors, but the difference was not significant (P = 0.143).

**Figure 2:**
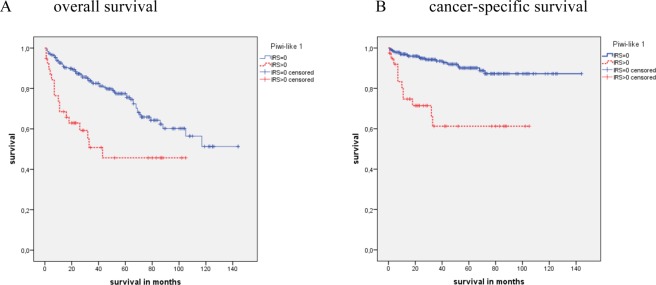
Kaplan-Meier analysis for OS and CSS of the subgroup of clear cell RCC patients in cohort 2. Patients with Piwi-like 1 positive tumors showed a significant association with shorter OS and CSS (both P < 0.001) compared to patients with Piwi-like negative tumors.

In cohort 2 (N = 274), 235 clear cell RCC patients (85.8%) were Piwi-like 1 negative, and 39 patients (14.2%) were Piwi-like 1 positive. The mean OS time for the Piwi-like 1 positive patients was 56.6 months vs. 101.7 months for the Piwi-like 1 negative patients (P < 0.001). Considering CSS, patients with Piwi-like 1 positive tumors had a mean survival of 69.9 months vs. 130.3 months for patients with Piwi-like 1 negative tumors (P < 0.001; Kaplan-Meier analysis Fig. 2). A univariate Cox’s regression analysis revealed that Piwi-like 1 positivity was associated with a 2.57-fold risk for death (P < 0.001) and a 5.10-fold increased risk for CSD (P < 0.001). A multivariate Cox’s regression analysis (adjusted for Fuhrman grade and tumor stage) confirmed that Piwi-like 1 positivity was an independent prognostic factor for OS (RR = 1.90; P = 0.023) and for CSS (RR = 3.19; P = 0.003). However, when the presence of distant metastases was included in the multifactorial model, tumor stage and Piwi-like 1 expression were no longer independent predictors of OS or CSS (data not shown).

#### Stratification according to Fuhrman grade and WHO tumor grade

Because of the correlation between Fuhrman grade and Piwi-like 1 protein expression, we tested if Piwi-like 1 positivity was a prognostic factor in the Fuhrman grade groups (grade1 + 2 or grade 3 + 4).

**Figure 3:**
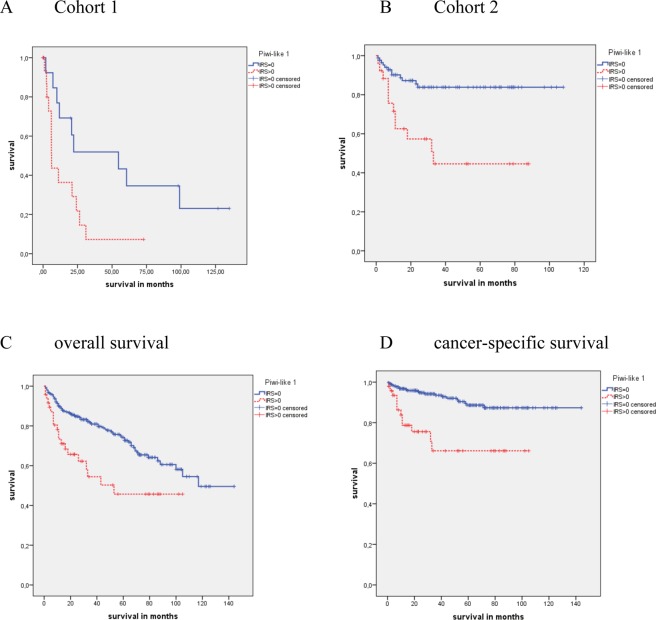
Kaplan Meier analysis for CSS survival in the Fuhrman grade 3 + 4 group in cohort 1 and cohort 2. Patients with Piwi-like 1 positive tumors possessed a significant association with shorter CSS in (**A**) cohort 1 and (**B**) cohort 2 (P = 0.024 and P < 0.001). Kaplan-Meier analysis for OS and CSS in the tumor grade 2 + 3 group in cohort 2. Patients with Piwi-like 1 positive tumors revealed a significant association with shorter (**C**) OS and (**D**) CSS (P = 0.002 and P < 0.001).

In cohort 1, only patients with higher Fuhrman grade tumors (3 + 4; N = 36) showed differences in OS and CSS associated with Piwi-like 1 positivity. The mean OS and CSS intervals for the patients with Piwi-like 1 positive tumors were 16.3 months and 16.0 months, whereas those patients with Piwi-like 1 negative tumors survived on average 52.1 months and 58.6 months, respectively. This difference was significant for CSS only (P = 0.056 and P = 0.024; Fig. 3A).

Likewise, in cohort 2, only patients with higher Fuhrman grade tumors (3 + 4; N = 113) showed differences in OS and CSS associated with Piwi-like 1 positivity. The mean OS and CSS intervals for patients with Piwi-like 1 positive tumors were 41.5 months and 46.9 months, whereas those patients with Piwi-like negative tumors had a mean survival of 65.0 months and 92.3 months. Again, the difference was significant for CSS only (P = 0.135 and P < 0.001; Fig. 3A).

Taken together, Piwi-like1 expression sub-stratified RCC patients with high Fuhrman grade tumors into groups with shorter or longer CSS.

Tumor grade according to WHO 2016^16^ was determined for cohort 2 only. In this cohort, the patients with a higher tumor grade (2 + 3, N = 303) showed differences in OS and CSS associated with Piwi-like 1 protein expression. The mean OS and CSS intervals for the patients with Piwi-like 1 positive tumors were 58.4 months and 74.5 months, whereas those patients with Piwi-like negative tumors survived, on average, for 99.5 months and 130.2 months (P = 0.002 and P < 0.001; Fig. 3B).

#### Stratification according to tumor stage

**Figure 4:**
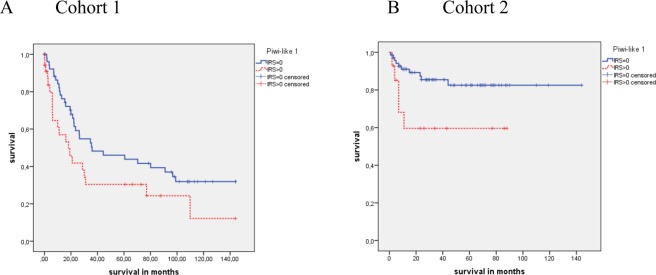
Kaplan-Meier analysis for CSS in the tumor stage T3 + T4 group in cohort 1 and cohort 2. Patients with Piwi-like 1 positive tumors possessed a significant association with shorter CSS in cohort 1 and cohort 2 (P = 0.044 and P = 0.027).

Since there was a correlation between tumor stage and Piwi-like 1 protein expression, we analyzed if Piwi-like 1 positivity was a prognostic factor in the tumor stage groups (T1 + T2 vs. T3 + T4) of the RCC patients. In cohort 1, a difference in the OS between patients with Piwi-like 1 positive tumors and patients with the Piwi-like 1 negative tumors was detected in the T3 + T4 group only, but this was not significant (N = 90; 42.9 vs. 66.7 months; P = 0.052). Again, a difference in CSS between the patients with Piwi-like 1 positive tumors and the patients with Piwi-like 1 negative tumors (N = 89; 43.9 vs. 67.5 months; P = 0.044; Fig. 4) was found in the T3 + T4 group only.

In cohort 2, there was a difference in OS between the patients with Piwi-like 1 positive tumors and the patients with Piwi-like 1 negative tumors in the T1 + T2 group (N = 250; 62.5 vs. 111.7 months; P < 0.001) but not in the T3 + T4 group (N = 85; 36.9 vs. 71.8 months; P = 0.176). An analysis of CSS between the patients with Piwi-like 1 positive tumors and the patients with negative tumors revealed significant differences in both tumor stage groups (T1 + T2 group: N = 250; 74.5 vs. 134.4 months; P < 0.001, and T3 + T4 group: N = 85; 55.0 vs. 121.7 months; P = 0.027; Fig. 4).

Altogether, in both cohorts, Piwi-like 1 expression separated patients with higher tumor stage (T3 + T4) into groups with shorter or longer CSS.

#### Stratification according to presence of distant metastases

We demonstrated a correlation between the presence of distant metastasis and Piwi-like 1 expression. In cohort 1 there was a difference in CSS between the patients with Piwi-like 1 positive tumors and the patients with Piwi-like 1 negative tumors with distant metastasis (M1; N = 56) (28.3 vs. 41.0 months), but this was not significant (P = 0.65).

**Figure 5:**
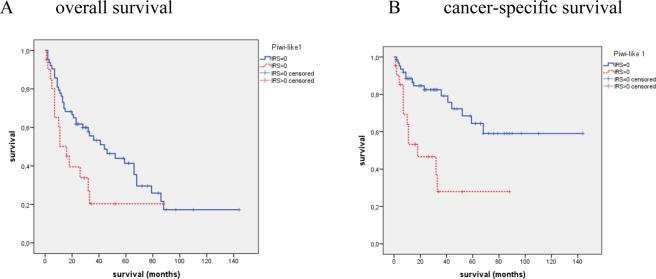
Kaplan Meier analysis for OS and CSS in patients with distant metastasis in cohort 2. Patients with Piwi-like 1 positive tumors showed a significant association with shorter OS and CSS (P = 0.035 and P < 0.001).

In cohort 2, only the patients that developed distant metastases (N = 84) showed differences in OS and CSS in association with Piwi-like 1 expression. The mean OS and CSS intervals for the patients with Piwi-like 1 positive tumors were 28.8 months and 35.0 months, whereas the patients with Piwi-like 1 negative tumors survived, on average, for 57.8 months and 98.9 months (P = 0.035 and P < 0.001; Fig. 5).

#### Stratification according to pre-operative C-reactive protein (CRP) levels

We previously showed that pre-operative CRP levels are an independent prognostic factor for OS and CSS in RCC patients^17^. We therefore separated the RCC patients in two groups (low: <5 mg/ml vs. high: ≥5 mg/ml). This cutoff was chosen in accordance with routine clinical diagnostics, where a threshold of 5 mg/ml is routinely used. Moreover, 5 mg/ml was also the median in cohort 1 (N = 256). As expected, patients with low pre-operative CRP levels had a mean OS interval of 115.6 months and a mean CSS interval of 118.6 months, whereas those with high pre-operative CRP levels showed an average OS of 65.5 months and a CSS of 72.6 months (both P < 0.001).

**Figure 6:**
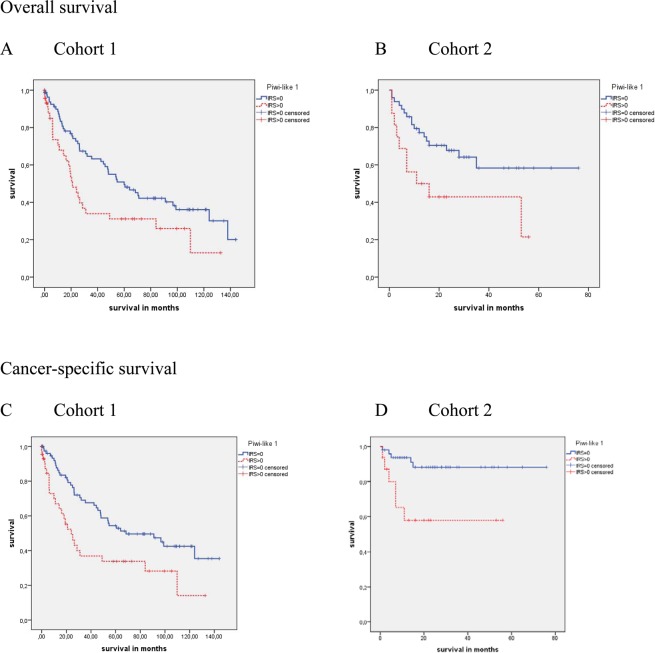
Kaplan-Meier analysis for OS and CSS in the CRP high group in cohort 1 and cohort 2. In cohort 1, patients with Piwi-like 1 positive tumors showed a shorter OS and CSS (P = 0.019 and P = 0.006). In addition in cohort 2, patients with Piwi-like 1 positive tumors revealed a shorter OS and CSS (P = 0.023 and P = 0.005).

In cohort 1, Piwi-like 1 expression was associated with the OS and CSS intervals only in the subgroup with higher CRP values (≥5 mg/ml). Hereby, patients with Piwi-like 1 positive tumors had a mean OS of 46.2 months and a mean CSS of 48.8 months, whereas those with Piwi-like 1 negative tumors showed, on average, an OS of 73.5 months and a CSS of 81.7 months (P = 0.019 and P = 0.006; Fig. 6).

In cohort 2, like before, the patients were separated into low and high pre-operative CRP level groups (low: <5 mg/ml vs. high: ≥5 mg/ml). The patients with low pre-operative CRP levels had a mean OS of 64.1 months. Notably, none of the patients with low pre-operative CRP levels suffered a CSD. The patients with high pre-operative levels showed a mean OS of 44.8 months, and eight patients died of CSD (both P < 0.001).

Stratified according to the defined CRP level groups, Piwi-like 1 expression was associated with differences in OS and CSS only in the high CRP group. Patients with Piwi-like 1 positive tumors had a mean OS of 26.7 months and a mean CSS of 34.8 months, whereas those with Piwi-like 1 negative tumors showed, on average, an OS of 50.4 months and a CSS of 68.0 months (P = 0.023 and P = 0.005; Fig. 6).

Altogether, Piwi-like 1 expression further sub-stratified the patient population with high pre-operative CRP levels into patients with a shorter OS and CSS (Piwi-like 1 positive) and patients with a longer survival (Piwi-like 1 negative).

## Discussion

More than 40,000 new cases of RCC are diagnosed in the European Union every year, and about half of these patients will eventually die from RCC. Since patients’ clinical courses vary and are difficult to predict, the stratifications of patients to appropriate postoperative surveillance programs and different therapeutic strategies tailored to the risk of cancer progression is increasingly important.

It is known that Piwi-like proteins bind together with small non-coding RNAs, so called piRNAs, as complex to DNA to recruit chromatin modifiers that enable transcriptional repression^18,19^. In this study, we investigated the relationship between Piwi-like 1 IHC and clinical parameters with a focus on OS and CSS in two RCC cohorts. A positive correlation of a Piwi-like 1 expression with Fuhrman grade, tumor stage and distant metastasis and a negative correlation with survival time were seen. In hepatocellular carcinoma, Piwi-like 1 protein expression is related to tumor size, lymph node and intrahepatic metastasis^20^. Furthermore, in cervical squamous cell carcinoma, Piwi-like 1 is correlated with tumor stage but not with lymph node metastasis^21^.

For the first time, we showed that Piwi-like 1 immunoreactivity was a negative prognostic factor for CSS in RCC. In the univariate Cox’ regression hazard analysis, Piwi-like 1 immunoreactivity (IRS > 0) was associated with a 1.59-fold (P = 0.033) and a 4.37-fold (P < 0.001) increased risk for CSD in cohort 1 and in cohort 2, respectively. In cohort 2 only, the multivariate Cox’s regression hazard model (adjusted to Fuhrman grade and tumor stage) showed that Piwi-like 1 immunoreactivity (IRS > 0) was associated with a 3.02-fold (P = 0.003) increased risk for CSD. Sun *et al*. reported that Piwi-like 1 protein expression is an independent prognostic factor for OS and CSS in colorectal cancer, but distant metastasis was not considered^22^. Furthermore, Zhao *et al*. showed that Piwi-like 1 protein expression was an independent prognostic factor for overall and relapse-free survival in hepatocellular carcinoma^20^. Interestingly, in their analysis, intrahepatic metastasis was of prognostic relevance only in the univariate but not in the multivariate analysis. However, when we included distant metastasis in our multivariate analysis, the formerly significant association of Piwi-like 1 positivity (and tumor stage) with CSS was not an independent prognostic factor any more.

Piwi-like 1 positivity in the tumor separated the patients with longer and shorter CSS within the high grade group and within the high tumor stage group. In addition, pre-operative CRP levels are associated with differences in OS and CSS in RCC patients^17^. In our study, Piwi-like 1 positivity separated the RCC patients with longer and shorter OS and CSS times in the group with high pre-operative CRP levels (≥5 mg/ml) in both cohorts.

However, how does Piwi-like 1 affect prognosis? Piwi-like 1 promotes the proliferation of colorectal cancer cells via upregulating global DNA methylation^23^, and it also translationally upregulates DNA methyltransferases (DNMTs)^24^. Piwi-like 1-associated DNA methylation is associated with the silencing of cyclin-dependent kinase inhibitors (CDKI), and the CDKIs p15, p21, and p27 show a tight IHC-based inverse correlation to Piwi-like 1 levels^24^. In line with the repression of CDKIs, as cell cycle controllers, is that the overexpression of Piwi-like 1 in MCF7 breast cancer cells increases the protein expression of cyclin dependent kinases (CDK 4, −6, −8) as cell cycle activators^25^.

Certainly, our study was limited by the subjectivity of the IRS determination and by its retrospective nature; therefore, further validation in prospective studies at other laboratories is necessary.

In summary, Piwi-like 1 protein expression is associated with a shorter CSS in patients with ccRCC. Piwi-like 1 positivity distinguishes between prognostic groups within patients who suffer from tumors of high Fuhrman grade, high tumor stage, distant metastasis and those with high pre-operative CRP levels.

## Material and Methods

### Patients and tumor material

Tissue microarrays (TMA) with formalin-fixed and paraffin embedded tumor samples with two cohorts of 265 (Hannover Medical School, cohort 1) and of 345 (Erlangen Medical Center, cohort 2) RCC patients that consisted of formalin-fixed and paraffin-embedded RCC tumors. Among both cohorts, clear cell histology dominated with 74.7 and 79.2% of cases, respectively. Observation time was 0 to 144 months and 1 to 144 months with a median of 62.1 and 38.0 months, respectively. The tumors had been resected and archived immediately after diagnosis for several years prior to TMA construction. The resection date range was between 1998 and 2011 for the Erlangen cohort and between 1996 and 2003 for the Hannover Medical School cohort, this is why the initial diagnosis was not comparable between cases and needed to be revised in accordance with the 2016 WHO classification on renal tumors^16^. The tumor histology was reviewed by experienced uropathologists (AH and Dr. M. Abbas/Institute of Pathology, Medical School Hannover). All the procedures were performed in accordance with the ethical standards established in the 1964 Declaration of Helsinki and its later amendments. All Erlangen patients beginning from 2008 gave informed consent. For samples before 2008 Ethic Commission in Erlangen waived the need for informed individual consent. The study is based on the approvals of the Ethic Commissions of the University Hospital Erlangen (No. 3755) and the University Hospital Hannover (No. 1741-2013/2351-2014). As we used archival tissue several years after resection, analysis of Piwi-like 1 protein expression was retrospective and did not affect clinical decisions. An overview of the clinico-pathologic parameters of the patients is given in Table 1. The levels of the C reactive protein (CRP) were determined by routine laboratory diagnostics (reference range < 5 mg/L).

### Immunohistochemistry

For the Piwi-like 1 protein expression study, a manual immunohistochemistry (IHC) protocol was applied as previously described^26^. Briefly, after heat pretreatment at 120 °C for 5 min with TE–buffer pH 9 and peroxidase blocking (Dako, Hamburg, Germany), a primary antibody against Piwi-like 1 (polyclonal goat IgG, N-17; Cat.-No. 22685; Santa Cruz, Heidelberg, Germany) was applied for 30 min. The stained specimens were viewed at an objective magnification of x100 and x200 by an experienced uropathologist (AH). The expression of Piwi-like 1 was detected in the cytoplasm by assessing the percentage of stained tumor cells and the staining intensity semi-quantitatively but it was not detected in the normal tissue adjacent to the tumor (Suppl. Fig. 1). Their product resulted in an immunoreactive score (IRS) from 0 to 12^27^. Negative control slides without the addition of primary antibody were included for each staining experiment. For the survival analysis, the patients were grouped by IRS = 0 and IRS > 0. Photos were taken with a Leica DM 4000B microscope with 20x HC PL Fluotar objective (Leica, Wetzlar, Germany) and with a Jenoptik Gryphax Arktur camera (Jenoptik AG, Jena, Germany). A white balance was performed.

### Statistical analyses

The correlations between the IHC and clinical data were calculated using a Chi^2^-test or Mann-Whitney test. The associations of the expression of Piwi-like 1 with the overall survival (OS) or cancer-specific survival (CSS) were determined by univariate (Kaplan-Meier analysis and Cox’s regression hazard models) and multivariate analyses (Cox’s regression hazard models, adjusted to Fuhrman grade and tumor stage) with follow-up periods censored at 12 years (144 months) to avoid non-tumor-related morbidity effects. Following standard practice in retrospective survival analysis, the common time point zero of all patients was the date of primary tumor surgery. A p-value < 0.05 was considered statistically significant. The statistical analyses were performed with the SPSS 21.0 software package (SPSS Inc., Chicago, IL).

## Supplementary information

Suppl. Fig.

Suppl. Table 1
