# Supplementary material for: Piwi-like 1 protein expression is a prognostic factor for renal cell carcinoma patients
Source: Sci Rep. 2019 Feb 11;9:1741. doi: 10.1038/s41598-018-38254-3 (PMC6370845; doi:10.1038/s41598-018-38254-3)
Supplement: Supplementary file 1 — Suppl. Fig. [file 41598_2018_38254_MOESM1_ESM.pdf]

Suppl. Fig. 1. IHC detection of Piwi-like 1

Piwi-like 1 protein expression is a prognostic factor for renal cell carcinoma patients

Christine G. Stöhr[1], Sandra Steffens[2,3], Iris Polifka[1], Rudolf Jung[1], Andreas Kahlmeier[4], Philipp Ivanyi[5], Florian Weber[6], Arndt Hartmann[1], Bernd Wullich[4], Sven Wach[4], Helge Taubert[4]

[1]Institute of Pathology, University Hospital Erlangen, FAU Erlangen-Nürnberg, Erlangen, Germany
[2] present address: Clinic for Urology, University Hospital Muenster, Muenster, Germany.
[3]Department of Urology, Hannover Medical School, Hannover, Germany
[4]Department of Urology and Pediatric Urology, University Hospital Erlangen, FAU Erlangen-Nürnberg, Erlangen, Germany
[5]Department of Hematology, Hemostasis, Oncology and Stem Cell Transplantation, Hannover Medical School, Hannover, Germany
[6]Institute of Pathology, University Regensburg, Regensburg, Germany

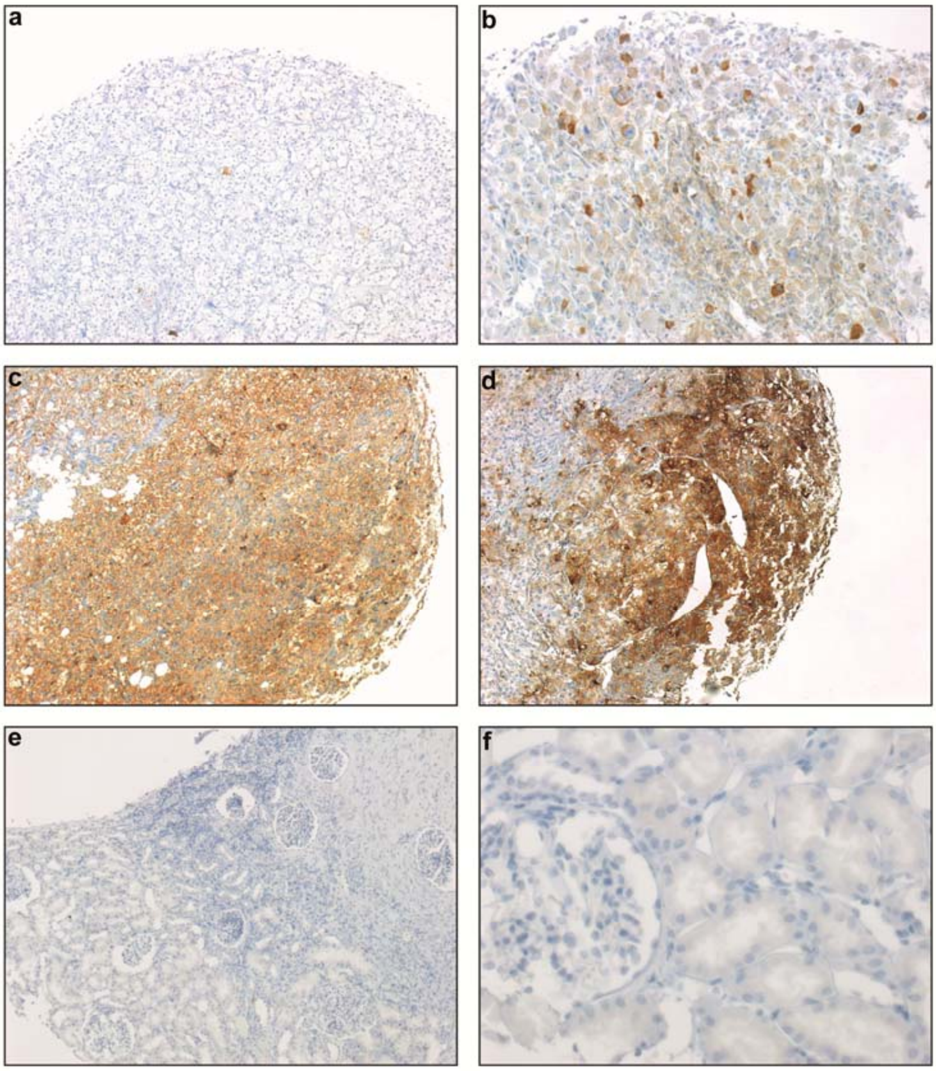

Immunohistochemical staining for Piwi-like 1 was assessed by an immoreactive score (IRS).

Piwi-like 1 staining in tumor cells with IRS=0 (a), IRS=4 (b), IRS=6(c), IRS=9 (d) and negative Piwi-like 1 staining in normal tissue adjacent to tumor cells (e,f). Photos are at a magnification of ×200 (a-d), ×100 (e) and ×400x (f).
