# Supplementary material for: Piwi-like 1 protein expression is a prognostic factor for renal cell carcinoma patients
Source: Sci Rep. 2019 Feb 11;9:1741. doi: 10.1038/s41598-018-38254-3 (PMC6370845; doi:10.1038/s41598-018-38254-3)
Supplement: Supplementary file 2 — Suppl. Table 1 [file 41598_2018_38254_MOESM2_ESM.docx]

**Suppl. Table 1 Piwi-like 1 expression in the two cohorts**

**Piwi-like 1 protein expression is a prognostic factor**

**for renal cell carcinoma patients**

Christine G. Stöhr^1^, Sandra Steffens^2,3^, Iris Polifka^1^, Rudolf Jung^1^, Andreas Kahlmeier^4^, Philipp Ivanyi^5^, Florian Weber^6^, Arndt Hartmann^1^, Bernd Wullich^4^, Sven Wach^4^, Helge Taubert^4^

^1^Institute of Pathology, University Hospital Erlangen, FAU Erlangen-Nürnberg, Erlangen, Germany

^2^ present address: Clinic for Urology, University Hospital Muenster, Muenster, Germany.

^3^Department of Urology, Hannover Medical School, Hannover, Germany

^4^Department of Urology and Pediatric Urology, University Hospital Erlangen, FAU Erlangen-Nürnberg,
 Erlangen, Germany

^5^Department of Hematology, Hemostasis, Oncology and Stem Cell Transplantation, Hannover Medical School,
 Hannover, Germany

^6^Institute of Pathology, University Regensburg, Regensburg, Germany

| **Piwi-like 1** | **Patients cohort 1 (%)** | **Patients cohort 2 (%)** |
| --- | --- | --- |
|  | 265 | 345 |
| IRS 0 | 190 (71.7) | 294 (85.2) |
| IRS 1 | 2 (0.7) | 0 |
| IRS 2 | 41 (15.5) | 28 (8.1) |
| IRS 3 | 0 | 2 (0.6) |
| IRS 4 | 28 (10.6) | 13 (3.8) |
| IRS 6 | 4 (1.5) | 3 (0.9) |
| IRS 9 | 0 | 5 (1.4) |
|  |  |  |
| IRS 0 | 190 (71.7) | 294 (85.2) |
| IRS>0 | 75 (28.3) | 51 (14.8) |
